# Nasal Septal Perforation closure with bacterial cellulose in rabbits

**DOI:** 10.1590/S1808-86942010000400007

**Published:** 2015-10-19

**Authors:** Eulógio Emílio Martinez Neto, Jose Eduardo Lutaif Dolci

**Affiliations:** a MD. MSc student - Otorhinolaryngology - Medical School of Santa Casa - São Paulo; b Full Professor of Medicine - Medical School of Santa Casa - São Paulo, Head of the Department of Otorhinolaryngology - Medical School of Santa Casa - São Paulo

**Keywords:** adhesive, fibrin tissue, surgery, cellulose, nasal septum

## Abstract

Alloplastic materials can be used together with tissue and structure to close nasal septal perforation.

**Aim:**

to test cellulose use in the closure of septal perforation in rabbits and to compare fibrosis, inflammation, vascular congestion and graft integrity.

**Materials and Methods:**

Fifteen rabbits. The rabbits were divided into two groups: Control: Five rabbits and Bionext® and fibrin glue Tissucol®: Ten rabbits. Septal perforations were done in all of them. In the Bionext® group the closure was performed with the placement of cellulose.

**Results:**

Two rabbits died in the first week. Cellulose group: 2 closures without the cellulose in between the septum membrane and in 4 cases the graft stood in the middle of the perforation locked in place by the edges. No closure in the control group.

**Conclusion:**

There was no closure of the perforation of the nasal septum with the graft between the septum membranes. There was no statistically significant difference concerning acute inflammation, vascular congestion and fibrosis between the 2 groups. In cases in which the graft remained in place, there was no change in its integrity. It may be used as a substructure for reepithelization of the perforation edges.

## INTRODUCTION

Nasal septum perforation is a disorder of easy diagnosis through the use of a nasal speculum or nasal endoscopy in the office. It can have numerous causes, from very benign ones, to local manifestations of systemic diseases. Moreover, nasal septum perforation varies broadly depending on location, size and symptoms; in consequence, many are the treatment options, including conservative treatment and numerous surgical techniques. Treatment must be customized for each patient.

The main cause of septal perforation is iatrogenic - as a complication of nasal surgery^1^^,^^2^; however, other iatrogenic causes may happen, such as the use of nasal steroids^3^, ^4^, ^5^, mucosal cauterization to treat epistaxis; nasotracheal intubation and turbinate cryosurgery. Moreover, nasal perforations have been described after many types of trauma surgery. Perforation rarely happens in children^6^.

Among the diseases associated with nasal perforation, we list: cutaneous mucosal leishmaniasis; nasal abscess; syphilis; tuberculosis; typhoid fever; diphtheria; Wegener granulomatosis; lupus erythematous and sarcoidosis. Neoplasia and carcinomas also can cause nasal septum perforation^7^, ^8^, ^9^. In tropical, developing areas, leprosy and Leishmaniasis still are among the causes of nasal septum perforation^10^^,^^11^. When the perforation borders are covered by mucosa, hardly the perforation is associated to more severe disorders, such as tuberculosis or neoplasias^3^^,^^1^.

Inhaling irritating products can also cause nasal septum perforation, such as in cases of nasal aspiration of cocaine, which causes ischemia by vasoconstriction^1^, foreign body granuloma caused by substances added to the drug3, besides perforations reported after inhaling chromic acid smoke, limo and cement powder, tar, pitch, salt, glass powder, sodium cabonate, calcium nitrate, calcium cyanide, arsenic, mercury and phosphorus.

Often times, the perforation happens before other systemic disease symptoms, and clinical investigation is necessary for those cases without clear etiological diagnosis^12^.

Nasal septum perforation can be range from asymptomatic to those causing severe epistaxis, nasal cosmetic deformities, crust forming and nasal obstruction^1^, ^2^, ^3^.

The surgeries proposed to close perforations use flaps and grafts. The latter can be autologous (removed from the same being), homologous (from another being of the same species), heterologous (from another being of another species) and alloplastic (synthetic materials). Autologous grafts have the main disadvantage of causing trauma to the donor areas; and homologous and heterologous grafts bear risk of contamination for the individual receiving tissue from another being^13^.

An inert material which could provide structure to the healing would be very useful in the treatment arsenal of this disorder. The cellulose film formed by the fermentation from Acetobacter xillinum bacteria is inert, resistant and insoluble, permeable to liquid and gas and resistant to stretching and traction. It is sterile, non-toxic and non-pyrogenic^14^.

Studies in rabbits, replacing the septal cartilage for cellulose showed a partial absorption of the cellulose film after four weeks^15^ and when used to cover the open area after nasal concha resection in rabbits, it proved tolerable to the receiving tissue and there was no difference as to the healing response concerning the control group^16^.

We propose an experimental study to close a nasal perforation in rabbits using the cellulose film together with fibrin glue used for fixation, and analyze the tissue response, graft properties and whether or not the perforation would be closed.

## LITERATURE REVIEW

Using the LILACS and Pubmed databases and the Internet, we searched the existing literature in relation to the cellulose produced by the Acetobacter xillinum bacteria, its development and use; surgical techniques used to close nasal septum perforation and the different materials used for this task.

Nasal septum perforation closure surgery in humans In 1935, Imperatori et al.^17^ proposed a surgical enlargement of the perforation, with the aim of reducing the discomfort brought about by constant hissing caused by smaller perforations, located on the anterior portion of the nasal septum.

The techniques used to close septal perforation can be grouped in: those in which we use flaps from the nasal septum itself to close it with or without grafts, and the techniques which use neighboring flaps.

The papers which suggest flap rotation are unanimous as to the need for a second surgical procedure to resect the vascular pedicle.

Among the techniques using septal mucosa flaps, we find the description of fascia and perichondrium grafting in between the septal mucosa leaflets, by Wright et al.^18^. This publication was greatly important, both because of the promising results, as well as for justifying the use of grafts to close the perforations, instead of simply suturing the borders. The authors also report that, besides the low metabolic requirements, fascia and perichondrium grafts serve as support for the growth of fibroblasts, supporting growth at the margins of the mucosas, one towards the other.

McCollough^19^ reported a successful closure of the septal perforation with ear-based graft. In 1980, Fairbanks^9^ described temporal fascia use with a diameter 2cm larger than the perforation to be covered. This same publication advocates that small perforations arising from surgery have a better chance of closure if done during the same surgical intervention, since the perforation diameter tends to increase during healing.

Fairbanks^8^ also spoke against surgical techniques which propose suturing the borders of the perforation, with or without everting the contralateral mucosa because of the atrophic and fragile characteristic of the tissue of the perforation borders. It also criticizes the techniques which use the oral mucosa, considering its non-transformation in ciliated tissue, thus having its drying as a consequence.

Suggestions concerning the use of artificial material or implants associated with closure techniques were given by Gyeney and Kerenyi^20^ who in 1977 reported the closure of septal perforation through fibrin implants (Bioplast®). Kridel et al. in 1998^21^ suggested the use of a biosynthetic substitute (Acellular Human Dermal Allograft®), having reported success in the closure of eleven among 12 perforations treated. The authors criticized the use of fascias, especially due to the need to use material to dry them, which causes early softening, making them difficult to handle. In cases of failure, there was a diameter reduction from 3cm to 5mm. In 2006, Lee et al.^22^ showed the use of fibrin glue and autologous cartilage grafts used to prevent nasal septum perforation during septoplasty.

Other materials such as gold, ivory, cobalt and cork have also been used^23^. Among the most used alloplastic materials we find those based on silicone, such as Silastic® which are largely used in facial structure surgeries thanks to their possibility of being sculptured and molded according to need. Although relatively inert from the biological standpoint, after its placement on the receiving site, a fibrous capsule can cover it with time, causing tangible mobility and consequently greater likelihood of migration and extrusion^24^.

Stoor et al.^25^ studied the septal closure and infection by Haemophilus influenza and Streptococcus pneumoniae with the use of bioactive glass (BAG) in 11 patients with septal perforation not having contamination by these germs in any patient and being able to close the perforation in 10 cases.

### The cellulose film

The Bionext® cellulose film arises from the fermenting of Acetobacter xylinum bacteria, done through the experiment carried out by microbiologist Luís Fernando Xavier Farah (1984) from Curitiba (PN).^26^

**Figure 1:**
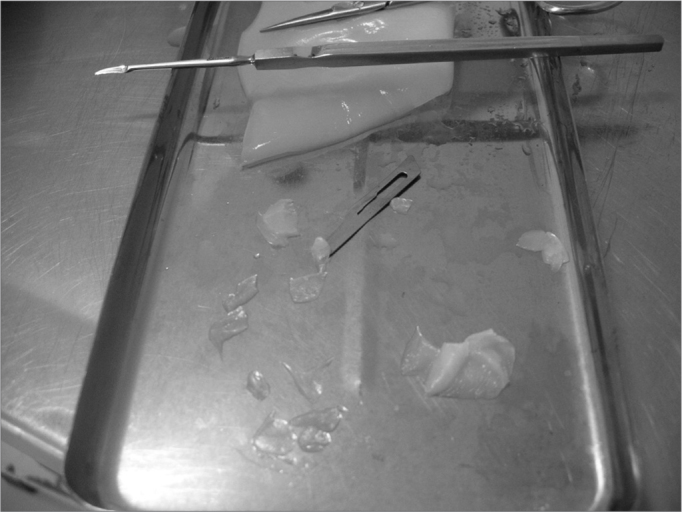
The cellulose mantle being sculptured.

The cellulose produced by the bacteria can be in the form of a flexible, semitransparent and yellowish membrane, or as a solid, dense, malleable mantle, of firm and jelly-like consistency, of about 0.5cm thick (Figure 1). The film arising from the biological production of the bacteria, after processing, does not have additives, being pure cellulose, made up of polysaccharides, biodegradable, non-toxic, non-pyrogenic and sterile. It is an inert substance, very resistant and insoluble in all organic solvents, and it has specific physical characteristics, such as: established permeability defined to liquids and gases, resistance to traction and stretching, also having characteristic weight and molecular structure.^14^^,^^27^^,^^28^

It is efficient for pain relief and to reduce healing time in dermoabrasion lesions, skin donor areas, or in large burns. This composite has been successfully used as bandage in skin sores, burns and skin donor areas. It was also used as substitute for meninges.^29^, ^30^, ^31^, ^32^, ^33^, ^34^

It is seen as a material of numerous possibilities, from paper to protect documents to bullet proof vests^26^. Acetobacter xilinum comercial cellulose, Bionext®, has been approved by ANVISA and the FDA and is used as a transitional skin replacement. Its physical and biocompatibility properties, as well as its ease of use and the possibility of modeling during insertion makes this substance a possible element in the treatment of bone and cartilage together.

## OBJECTIVES

1 To test the use of cellulose produced by Acetobacter xylinum bacteria -Bionext® together with the Tissucol® biological fibrin glue in the closing of septal perforation surgically inflicted in rabbits.
2 To histologically compare the degree of fibrosis, vascular congestion in the animals, graft integrity and whether or not the septal perforation was closed.

## MATERIALS AND METHODS

### Materials

For this study we used fifteen adult New Zealand rabbits weighing approximately 3kg, cellulose mantle (Bionext®) and fibrin biological glue (Tissucol®).

Sample size and selection

The rabbits were randomly assigned (coin flip) into two groups: Control Group made up of five rabbits; and the Bionext® group associated with the fibrin glue Tissucol®, made up of 10 rabbits.

One rabbit from each group died in the immediate post-op. the rabbits were slaughtered fifty days after the experiment.

### Surgical procedure

The surgical procedures were carried out following the ethical principles of experimentation with animals, set forth by the Brazilian Code of Animal Experimentation (COBEA). Protocol 09/07 ICAO.

The surgical procedures were done with the rabbits under general anesthesia, using Zoletil® (Tiletamine associated with Zolazepan) and Nilperidol® (Fentanyl associated with Droperidol) and kept under spontaneous ventilation, in dorsal decubitus. They were submitted to nasal septum perforation as per described below:

1 2.2% lidocaine and noradrenaline soaked cotton in the concentration of 1:50,000 in both nasal cavities.
2 Bilateral subperichondrial septum injection of the same solution.
3 Upper left-side anterior septal incision followed by ipsilateral subperichondrial detachment.
4 Square-shaped incision and removal of approximately 7mm measured with a surgical caliper, on each side of the septal mucosa and septal cartilage.

**Figure 2:**
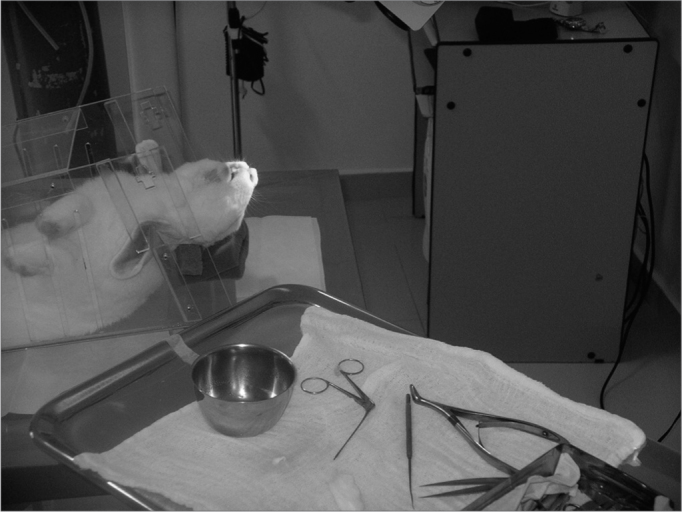
Rabbit positioned and anesthetized for the procedure.

The experimental group was submitted to the placement of the Bionext® graft in the same procedure when the perforation was made; anchored in between the remaining septal mucosa, which were previously detached and not removed (Fig.2).

The rabbits were kept under the same daily care conditions as they were prior to treatment, for 50 days, when they were slaughtered for observation of the nasal septum perforation and underwent histopathology analysis of the surgical specimen.

For the slaughter at the end of the follow up period, the rabbits were again anesthetized and received IV sodium thiopental (dose 40mg/kg). After the slaughter, the facial mesostructure was removed en bloc for histopathology purposes.

The rabbits were evaluated by clinical parameters which could indirectly assess tolerability issues, such as general and breathing discomfort. The parameters evaluated were: amount of food ingested, rabbit weight variation, temperature, respiratory rate and the presence of nasal bleeding. All the rabbits were weighed before the procedure and on a daily basis until their slaughter. The amount of food ingested was controlled daily in grams. Ear temperature, in Celsius was measured twice a day. Respiratory rate was checked twice-a-day. Bleeding was seen daily.

The animals' facial mesostructures were dissected and fixed in a 10% formaldehyde solution.

### Histological evaluation

**Figure 3:**
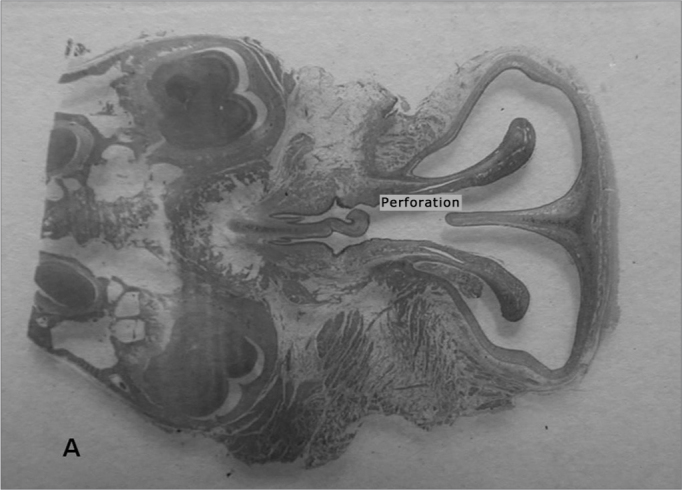
Photomicrography of septum histology slides. A - Ruptured septum (macro). HE dye.

The animals' facial mesostructures remained for 6 days in a 5% nitric acid solution for decalcification. Once decalcified, the nose and skull were sliced in 5mm cross-sections. The slices were then dehydrated, clarified and included in paraffin. After inclusion in the paraffin blocs, the specimens were cut in the microtome at an average thickness of 5μm. (Figure 3)

**Figure 4:**
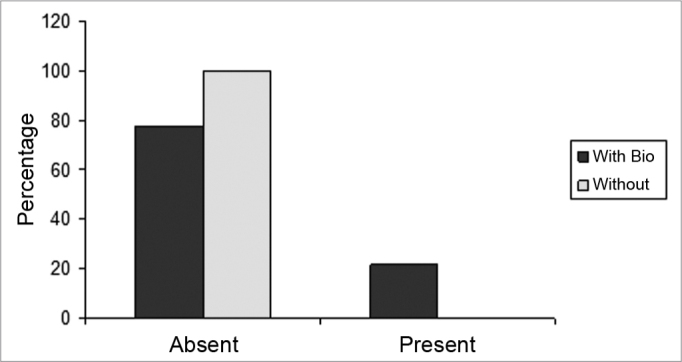
Percentage of animals that presented or did not present biomaterial integrity, in the control group (without) and experimental group (with Bio). (Fisher test: p=0.462).

**Figure 5:**
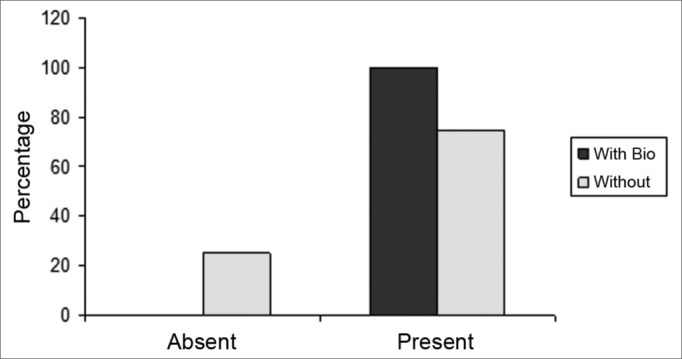
Percentage of animals who had or did not have congestion, in the control (without) and experimental (with bio) groups. (Fisher test: p=0.308).

**Figure 6:**
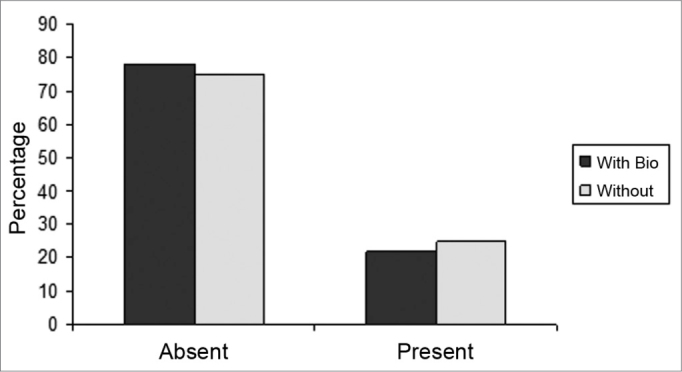
Percentage of animals who had or did not have an acute inflammatory process, in the control (without) and experimental (with bio) groups. (Fisher Test: p=0.706)

The slices were then dyed with hematoxylin-eosin and the tissue response patterns were histologically assessed. We analyzed: whether or not there was an inflammatory process; Bionext® (ibt) integrity; fibrosis; congestion and whether or not the septum perforation had been closed (Table 1). The slides were analyzed by only one pathologist in a blind way as to germ group and species, and classified in degrees by qualitative and semiquantitative criteria. Thus, Bionext® (IBT) integrity was classified in: 0=not applicable/ Bionext® absent, 1= intact and 2= fragmented (Figure 4); vascular congestion: 0= absent, 1= mildly congested, 2= dilated and congested, and 3= highly dilated vessels with red blood cell extravasation (Fig. 5); Fibrosis: 0=absent, 1= presence of fibroblasts alone and 2= reparative fibroblastic proliferation with thickness; acute inflammation (AI): we also looked for fibrin and neutrophilic exudate: 0=absent, 1= scattered spots and 2= neutrophil build up involving the structures (Fig. 6).

**Table 1:** Distribution of the rabbits as to the treatment given, weight (g) and histopathology parameters analyzed.

Rabbit	With Bionext	W/out	Weight pre	Weight post	bti	ai	Cong	fibrosis	Septum
1	x		5100	6200	0	0	2	0	Ruptured
2		X	4080	4237	0	0	3	0	Ruptured
3	x		3517	3691	0	0	2	0	Closed
4	x		4167	4180	0	0	1	0	Closed
5		X	4157	4238	0	0	2	0	Ruptured
6		X	3438	3751	0	0	0	0	Ruptured
7		X	3570	3693	0	2	3	0	Ruptured
8	x		3150	3774	0	0	3	0	Ruptured
9	x		3305	3508	1	1	1	0	Bt
10	x		3210	3109	0	0	2	0	Ruptured
11	x		3200	3422	0	0	2	0	Closed
12	x		3230	3247	1	1	2	0	Bt
13	x		3185	3392	0	0	2	0	Closed

bti =biotissue integrity (0=not applicable/ Bionext® absent, 1= intact and 2= fragmented); ai= acute inflammation (fibrin and neutrophilic exudate: 0=absent, 1= scattered spots and 2= neutrophil build up involving the structures); cong= vascular congestion:(0= absent, 1= mildly congested, 2= dilated and congested and 3= vessels highly dilated with red blood cell spill over); fibrosis: (0=absent, 1= scattered fibroblasts and 2= reparative fibroblastic proliferation with thickening) and septum (Bt= Bionext® between the perforation borders)

### Statistical Assessment

We compared the results from the Control and Bionext® groups by means of the F test of independent samples with non-paired comparisons between the groups as a whole. For parameters with discontinuing categorical values, such as the degree of inflammation, we used the Wilcoxon non-parametric test.

## RESULTS

Two rabbits died in the first week of the experiment, one from the Bionext® group and one from the Control Group.

On Table 1 we see the parameters assessed from each rabbit, according to their weight in the beginning of the experiment and after treatment, and the histological characteristics assessed on the specimens analyzed.

On Table 2 we see the data regarding the graft behavior and whether or not the septal perforation was closed.

**Table 2:** Histological changes seen in relation to the integrity of the biomaterial and characteristics of the septum, according to the group studied.

Groups	n	BTI			SEPTUM	
		0	1	Ruptured	Bionext®	Closed
With Bio	9	7	2	3	2	4
W/out	4	4	0	4	0	0

Bionext®: Cellulose present between the borders of the perforation We did not notice statistically significant difference between the groups as to biomaterial integrity (BTI) (p=0.462) and septum closure (p=0.176).

## DISCUSSION

Because of the need for a framework for fibroblasts to grow - supporting growth at the margins of the mucosa, one towards the other through a simple border suturing^18^^,^^2^, we considered the possibility of using a cellulose film placed between the borders of a surgical perforation done to the nasal septum of rabbits in order to serve as a support for a possible tissue regeneration.

Because the perforation and repair surgery were carried out in the same procedure and with the film already in place since the onset of the healing process, we did not have atrophic margins and we had very little vascularization, which is common in the clinical practice and does not foster closure^8^. This is a bias in our study.

Knowing of the ease in intraoperative handling, although of little adhesiveness, we added biological glue to the margins of the perforation with the aim of fixing the film. Thus, the experiment would be similar to the one from Kridel et al., who in 1998, who suggested the use of biosynthetic substitute (Acellular Human Dermal Allograft®) fixed with suture points to one of the sides and the other one is closed with mucosa flap rotation; nonetheless, in our case we used cellulose film and biological glue without closing the mucosa in any of the sides of the perforation.^21^

The film incorporation and absorption were more significant after four weeks in the studies by Oliveira^15^ studying the replacement of the septal cartilage for cellulose film. For this reason we chose to assess our sample after 50 days.

All the studies carried out evaluating the cellulose film had it in contact with some tissue, we found it interesting to assess its behavior when it is attached only to the borders of the perforation.

**Figure 7:**
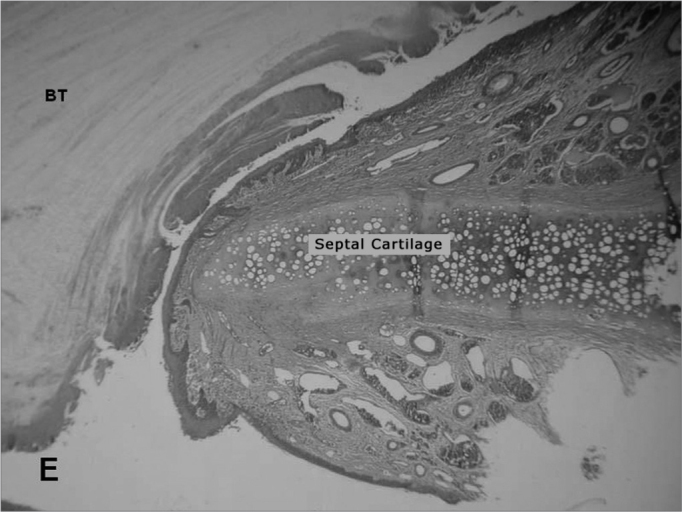
Photomicrography of septum histology slides: E=Ruptured septum and Bionext® (10x); BT= Bionext® HE dye.

**Figure 8:**
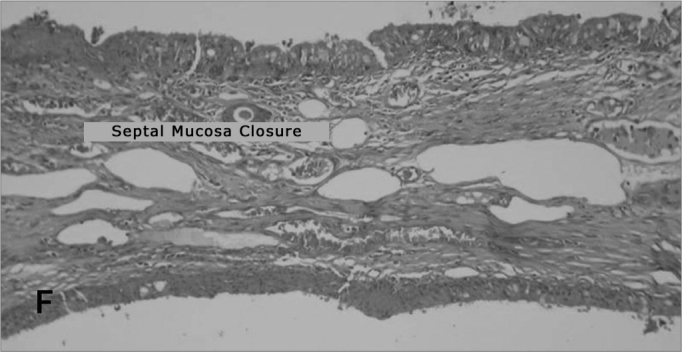
Microphotography of histological slides of the septum F = Intact reepithelized septum. (10x). HE dye.

The perforation closure with the cartilage replacement for the cellulose did not happen. In the cases in which the perforation closed, either the cellulose film was kept attached to the perforation's epithelized borders like a “cork” (Fig. 7), or it was lost very likely due to graft shifting. In the cases in which the closure happened without the graft (Fig. 8), the question remains whether or not the graft helps close the perforation, and at what time the shifting happened. Despite the lack of statistical significance, we can suspect the film helped in the closure since the control group did not show any closure of the perforated septa; and such significance was due to the small number of rabbits in the sample.

The graft shifting may have happened because of the smooth surface of the cellulose film, and due to the lack of efficacy of the fibrin glue in contact with the cellulose or by the mechanical force of sneezes. Postoperative packing for a long period of time, such as the 12 days proposed by Kratz in 1973^35^ or for shorter periods such as the two days proposed by Lee in 2008^36^ is not feasible in rabbits with a delicate graft on the nasal septum.

The lack of statistically significant difference as far as acute inflammation, vascular congestion and fibrosis are concerned, prove that the implant is inert and well accepted by the nasal mucosa.

The weight gain of the group submitted to the graft shows it is well tolerated by the animals and also shows little suffering with the experiment.

## CONCLUSIONS

a) There was no statistically significant difference concerning acute inflammation, vascular congestion and fibrosis between the two groups.
b) In the cases in which the graft was kept positioned, there was no change to its integrity.
c) It can be useful in the therapeutic weaponry as a basis for reepithelization of the perforation borders.
